# Intergenic and Genic Sequence Lengths Have Opposite Relationships with Respect to Gene Expression

**DOI:** 10.1371/journal.pone.0003670

**Published:** 2008-11-07

**Authors:** Juliette Colinas, Scott C. Schmidler, Gil Bohrer, Borislav Iordanov, Philip N. Benfey

**Affiliations:** 1 Department of Biology and IGSP Center for Systems Biology, Duke University, Durham, North Carolina, United States of America; 2 Department of Statistical Sciences, Duke University, Durham, North Carolina, United States of America; 3 Department of Civil & Environmental Engineering & Geodetic Science, Ohio State University, Columbus, Ohio, United States of America; 4 Hollywood, Florida, United States of America; Centre de Regulació Genòmica, Spain

## Abstract

Eukaryotic genomes are mostly composed of noncoding DNA whose role is still poorly understood. Studies in several organisms have shown correlations between the length of the intergenic and genic sequences of a gene and the expression of its corresponding mRNA transcript. Some studies have found a positive relationship between intergenic sequence length and expression diversity between tissues, and concluded that genes under greater regulatory control require more regulatory information in their intergenic sequences. Other reports found a negative relationship between expression level and gene length and the interpretation was that there is selection pressure for highly expressed genes to remain small. However, a correlation between gene sequence length and expression diversity, opposite to that observed for intergenic sequences, has also been reported, and to date there is no testable explanation for this observation. To shed light on these varied and sometimes conflicting results, we performed a thorough study of the relationships between sequence length and gene expression using cell-type (tissue) specific microarray data in *Arabidopsis thaliana*. We measured median gene expression across tissues (expression level), expression variability between tissues (expression pattern uniformity), and expression variability between replicates (expression noise). We found that intergenic (upstream and downstream) and genic (coding and noncoding) sequences have generally opposite relationships with respect to expression, whether it is tissue variability, median, or expression noise. To explain these results we propose a model, in which the lengths of the intergenic and genic sequences have opposite effects on the ability of the transcribed region of the gene to be epigenetically regulated for differential expression. These findings could shed light on the role and influence of noncoding sequences on gene expression.

## Introduction

**Figure 1:**
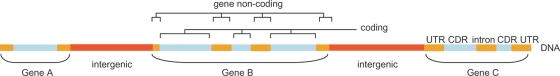
Schematic of the DNA sequences associated with a protein coding gene. The thick line represents double stranded DNA, orange-red for intergenic, dark blue for coding regions (CDR), and light blue for gene noncoding (introns, and UTRs at the ends of the gene). Depending on the orientation of the genes flanking it, an intergenic region can be either upstream of both surrounding genes, downstream of both, or upstream of one and downstream of the other (not shown here).

‘Noncoding DNA’ can be found both surrounding genes, and within genes (see schematic Figure 1). We will call the first type ‘intergenic’, and the second type ‘genic’, a ‘gene’ referring here to a transcribed DNA sequence. Intergenic DNA can be in front of the gene, i.e. upstream intergenic, or at the end of the gene, i.e. downstream intergenic. In the upstream sequence, there is the core promoter and various other regulatory elements. This upstream sequence is thought to be involved in recruiting the transcriptional machinery, for production of a mRNA transcript. The regulatory function of the downstream sequence is less well understood. The gene itself is composed of three different types of sequences: untranslated regions (UTRs) at the ends, and zero or more introns and one or more coding regions inside. After transcription, the process of splicing removes the introns from the transcript. The remaining transcript is composed of exons, each terminal exon containing a UTR. The coding regions (exons minus the UTRs) will be translated into protein. Introns and UTRs sometimes affect mRNA production either prior to transcription via their regulatory element content, or during and after transcription via microRNA mediated mRNA degradation, and other mechanisms [1]–[3]. However, contrary to the upstream intergenic sequences, introns and UTRs have not been ascribed a general role in regulating gene expression.

Over the past few years, studies in both plants and animals have shown correlations between the length of the intergenic and genic sequences of a gene and the expression of its corresponding mRNA transcript. Some studies have focused on the relationship between intergenic sequence length and diversity of expression level across tissues, and found that it was positive [4]–[6]. The general interpretation, based on the current understanding of gene regulation, is that genes under greater regulatory control require more regulatory information, resulting in a longer upstream intergenic sequence [4], [6]. However this interpretation does not explain why a positive relationship is also observed for downstream intergenic sequences [6], if, as a recent study with 61 *Arabidopsis thaliana* transcription factor genes suggests, the downstream intergenic sequence is generally not required to drive the appropriate gene expression pattern [7]. Other studies have focused on gene rather than intergenic length, and found that there is a negative relationship between gene length and expression level. This was usually interpreted as a sign of selection pressure for highly expressed genes to remain small [8]–[10]. Other explanations have been proposed, such as “transcriptional interference”, whereby highly expressed genes would tend to be more distant from adjacent genes such that their transcription is not hindered by that of their neighbors [11]. Moreover, reports have shown that there is also a correlation between gene length and expression pattern [6], [12]. The reason for this remains unclear, since it cannot be explained easily by our current understanding of gene expression regulation [6], [12]. In any case, because different studies use different datasets and measures of gene expression, it is difficult to draw from them a clear picture of the relationships between sequence length and gene expression.

Here, we sought to study the relationships between sequence length and mRNA expression of protein-coding genes, thoroughly and without *a priori* hypotheses. Using nonparametric smoothing regression we studied the relationships between intergenic, genic coding and genic noncoding sequence length, and three different aspects of gene expression across tissues. For our expression data, we made use of tissue-specific global gene expression data of high resolution from the root of the plant *A. thaliana* [7], [13]–[15]. To ‘measure’ gene expression, we sought to capture measures of gene expression that would relate not only to “level” and “breadth” (or “diversity”) of expression across tissues, but also to biological noise (i.e. random variation) which has been shown in recent years to be an important component of gene expression [16], [17]. For expression level we used the median expression across tissues; for the expression breadth or pattern, the variability between tissues; and for noise, the variability between biological replicates.

We found that intergenic and genic sequences have opposite relationships with respect to both expression variability and expression level, and that this does not hold for coding sequences when considered individually. Moreover, we found similar results for expression variability and for noise. Finally, categories of genes expressed with greater variability generally have longer intergenic sequences and shorter gene noncoding sequences, but not as much difference is observed for the coding sequence. To explain these results we propose a model, in which the length of intergenic and genic sequences have opposite effects on the ability of a gene to be epigenetically regulated for differential expression.

## Results

With the goal of precisely identifying genome-wide interdependencies between the length of the sequences associated with a gene and the expression of its mRNA transcript, we used the genome sequence of *A. thaliana* and gene expression data derived from microarray experiments. Before comparing sequence lengths and gene expression, we first determined if interdependencies existed between the different sequences themselves, and between the measures of transcript expression that we used.

### Interdependencies between genetic sequences

We first separated the sequences associated with a gene into four entities: 1) upstream intergenic, 2) downstream intergenic, 3) coding (the sum of all the coding regions) and 4) gene noncoding (introns and untranslated regions (UTRs)) (Figure 1). We did not separate intergenic sequences based on the orientation of their flanking genes (which could be either the same or opposite) because we did not observe any substantial effect for this factor on their relationship with gene expression (not shown).

**Figure 2:**
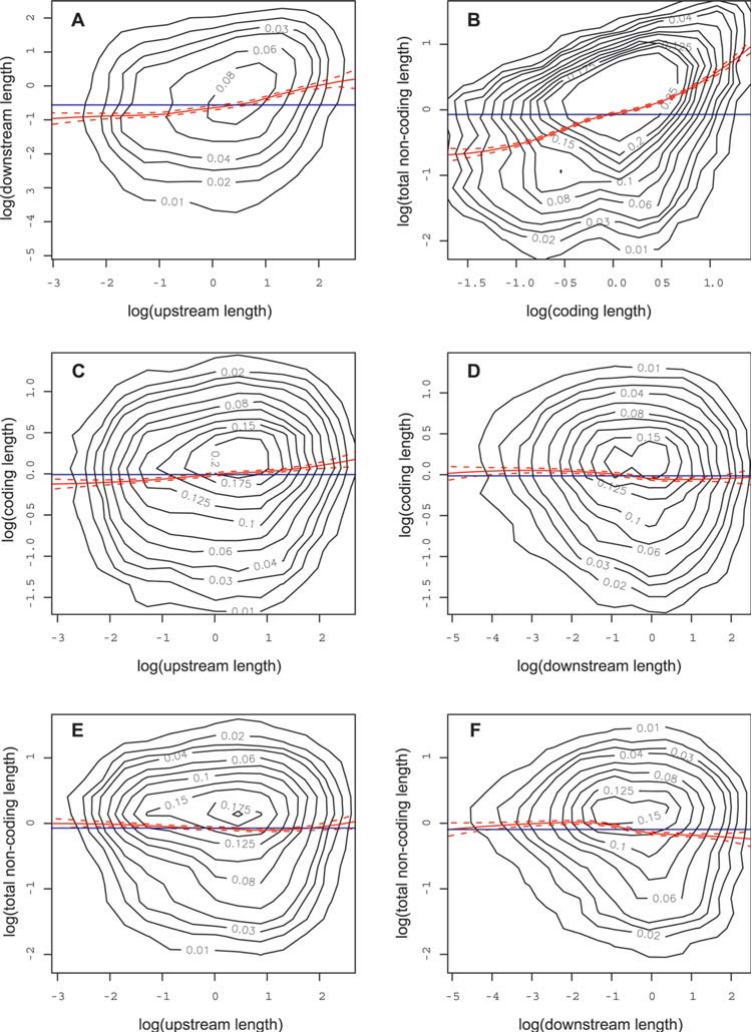
Inter-dependencies between the sequence lengths associated with a gene. Relationships between the natural logarithm of lengths (in kb) of the sequences associated with a gene. For this figure and Figure 3–5, the contours were obtained from a 2 d kernel density estimate. The numbers on the contours show the cumulative density contours (the total probability contained within the contour). The solid red line shows the trend in y axis as a function of x axis obtained via a local linear regression smoother. Dashed red lines show 95% confidence intervals, blue lines show constant mean and axes are scaled to the (0.5%, 0.95%) quantiles of the respective variables.

We found a positive relationship between the upstream and the downstream intergenic sequence lengths (Figure 2A), and a stronger (i.e. regression line of greater slope) positive relationship between the coding and gene noncoding sequences (Figure 2B). However, the relationships between intergenic and genic sequences were weak (Figure 2C, D). These results should be kept in mind as we describe the relationships between sequence length and gene expression.

### Expression level, variability between tissues and noise are inter-dependent

We chose to consider the three aspects of gene expression that have generally been considered by others: 1) overall expression level in a group of tissues (e.g. [8]–[10]); 2) some measure of the unevenness of the expression pattern across tissues [4]–[6]; and 3) expression noise [16], [17] which has not been previously studied in the context of relationships with sequence lengths. To obtain values for these three metrics, we used tissue-specific genome-wide gene expression data from different tissues of the root of *A. thaliana* [7], [13]–[15]. Comparisons of these data to other experimental results suggest that they provide a reliable estimate of the expression pattern of the mRNA [7], [13]–[15]. For level we used the **median** across-tissue expression; for the expression pattern we used the **variability of the expression across tissues** (a more variable expression is likely to represent a more tissue-specific expression pattern); and for **noise** we used the variability between biological replicates, i.e. between groups of plants grown independently (see Methods for precise definitions). This noise measure should include noise from both technical and biological origin. Because we do not expect technical noise to be related to sequence length (especially in the case of intergenic sequence length, which is unrelated to the microarray method of assaying mRNA level), any relationship between noise and length should presumably arise from the biological noise component.

**Figure 3 pone-0003670-g003:**
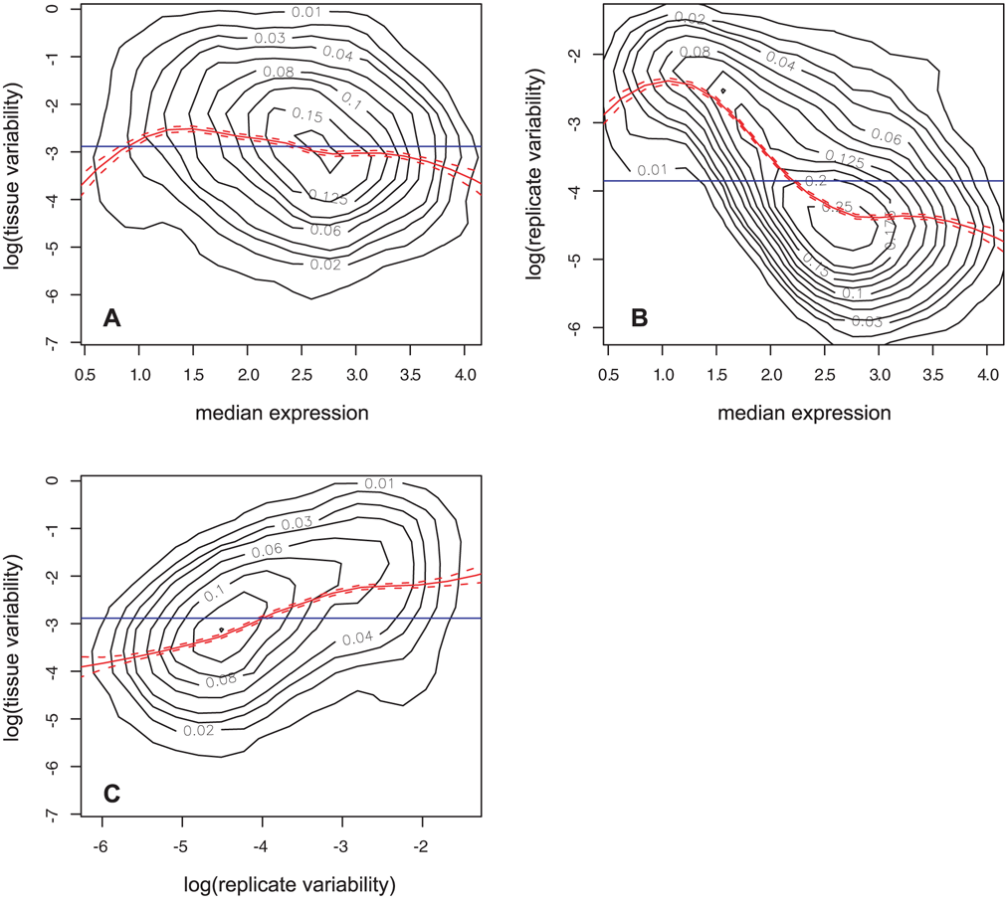
Inter-dependencies between inter-tissue median, and inter-tissue variability, and noise of expression. Relationships between our gene expression measures for expression level (median across tissues), variability (log of variability between tissues) and noise (log of variability between biological replicates).

We investigated the relationships between our three measures of expression. Tissue variability and noise had a generally negative but non-linear relationship with median expression, but it was much stronger for noise than for tissue variability (Figure 3A, B). Tissue variability and noise had a generally positive relationship (Figure 3C). These results should also be kept in mind as we describe below the relationships between sequence length and expression.

### Intergenic and genic sequence lengths have opposite relationships with respect to expression

**Figure 4 pone-0003670-g004:**
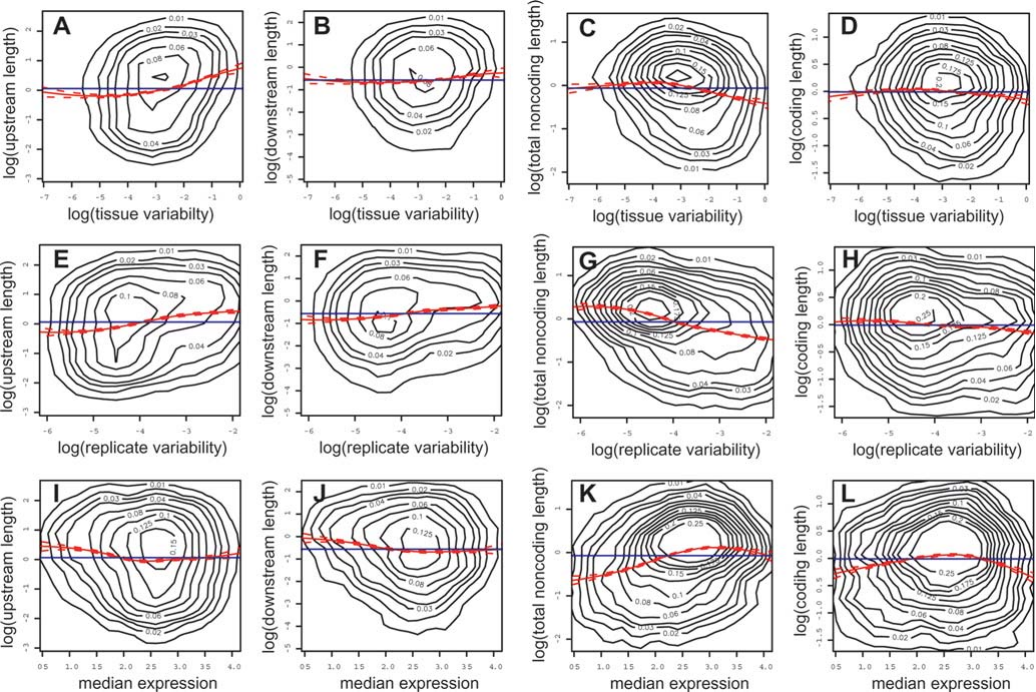
Upstream and gene coding or noncoding sequence lengths have opposite relationships with mRNA expression. Relationships between the (log) lengths (in kb) of the sequences associated with a gene and its diagnostic expression measures for variability (tissue variability), noise (replicate variability) and level (median).

We then studied the relationships between sequence length and mRNA expression for the 11,725 genes for which both types of data were available (see Methods). For **variability between tissues**, we found that more variable expression was associated with longer intergenic sequences [4]–[6] (Figure 4A, B), and with shorter genic sequences (Figure 4C, D). A negative relationship between variability and gene sequence length has been reported in a previous *A. thaliana* study [6], but we note that the relationship is stronger for the genic noncoding than it is for the coding sequence (Figure 4C vs. D). For **noise**, the relationships were similar to those with variability, and they appear even stronger than those with variability in general (Figure 4E–H). Also, for noise the relationship with the gene noncoding sequence is clearly much stronger than it is with the coding sequence (Figure 4G vs. H). For **median** expression, the relationships were not as linear, but they were globally negative for the intergenic sequences, and globally positive for the genic noncoding sequences (Figure 4I–K). However, there was a tendency for levels above and below the mean to be associated with shorter genic sequences and with longer intergenic sequences, except for the downstream sequence for which the relationship with median levels above the mean is very weak (Figure 4I–L). Contrary to the case for noise and variability, the coding sequence had a relationship with median expression similar to that of the noncoding sequence, but it was more symmetrical than for the noncoding sequence (Figure 4L).

These relationships should be viewed in light of the inter-dependencies between the variables themselves. First, we found that the gene coding and noncoding sequences have similar relationships to expression, although they are weaker for the coding sequence (Figure 4). This could either result from their interdependency (Figure 2B), or from both the gene coding and the gene noncoding sequences having a direct relationship with expression. In the first case, one of them wouldn't have a direct relationship, but a relationship only due to its dependence on the other. For example, it is possible that the length of the coding and of the noncoding sequences are strongly related to each other because of structural constraints imposed by the process of splicing [18], such that the coding sequence length only has a relationship with expression because it is tightly tied to the gene noncoding sequence. This is suggested by the fact that the coding sequence has weaker relationships with variability and noise than the gene noncoding sequence does. However, in the case of median expression, the coding sequence and the noncoding sequence have relationships of similar strength. Thus it could be that multiple factors are at play and that the coding sequence and noncoding sequences are not related to the different measures of expression for the same reasons. Second, we found that the upstream and downstream intergenic sequences have similar relationships with expression, however they are also positively inter-related (Figure 2A), so it could be that the weaker relationship of the downstream sequence with expression is a result of its relationship with the upstream sequence, due to other reasons, or that both have a direct relationship with expression. However we do not know of mechanisms which would constrain the downstream and upstream sequence lengths relative to each other. Finally, we note that the sets of relationships of each of the three different expression variables are consistent with the inter-dependencies between the expression variables themselves (Figure 3), so that we cannot differentiate the different expression variables.

Overall therefore, we conclude that intergenic sequences (upstream or downstream) globally have relationships with gene expression that are the opposite of those of the genic sequences (noncoding or coding) (Figure 4). This cannot be due to inter-dependencies between intergenic and genic sequence lengths since they are very weakly related to each other (Figure 2C, D).

### Individual coding and gene noncoding sequences have opposite relationships with variability and level

**Figure 5:**
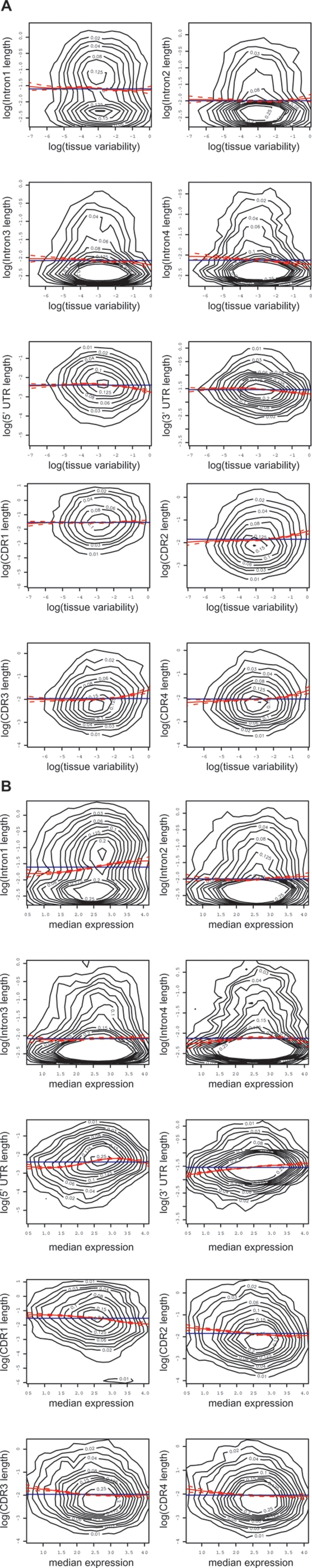
Gene noncoding sequence components, but not coding, maintain the negative relationship with variability. Relationships between the (log) lengths (in kb) of the genic sequences associated with a gene and (A) expression variability (tissue variability) and (B) level (median). CDR: coding region. We considered only the first four introns and coding regions of a gene.

We then examined the individual components of the genic sequences, i.e. the individual coding regions, introns, 5′ and 3′ UTRs, limiting ourselves to the first four introns or coding regions, and to tissue variability and median expression. For **tissue variability**, all components had very weak relationships to variability. However, although weak, the relationships were negative for the noncoding components (introns and UTRs), and positive for the coding components (Figure 5A). For **median expression**, although the relationships are stronger than they are for variability, again they are opposite for gene noncoding and coding components, globally positive for the first, and globally negative for the second (Figure 5B). Thus overall, gene noncoding and gene coding sequence components studied individually have relationships with tissue variability and median expression that are the opposite of each other. This opposite relationship could not be seen when looking at the entire coding and gene noncoding sequences (Figure 4).

Therefore the individual components of the gene noncoding sequence have similar relationships with expression as the total gene noncoding sequence does. However this is not the case for the gene coding sequence. This argues for the possibility that the relationships of the total coding sequence with expression are indirect and caused by its strong correlation to the total gene noncoding sequence.

### The trends hold for individual examples

**Figure 6:**
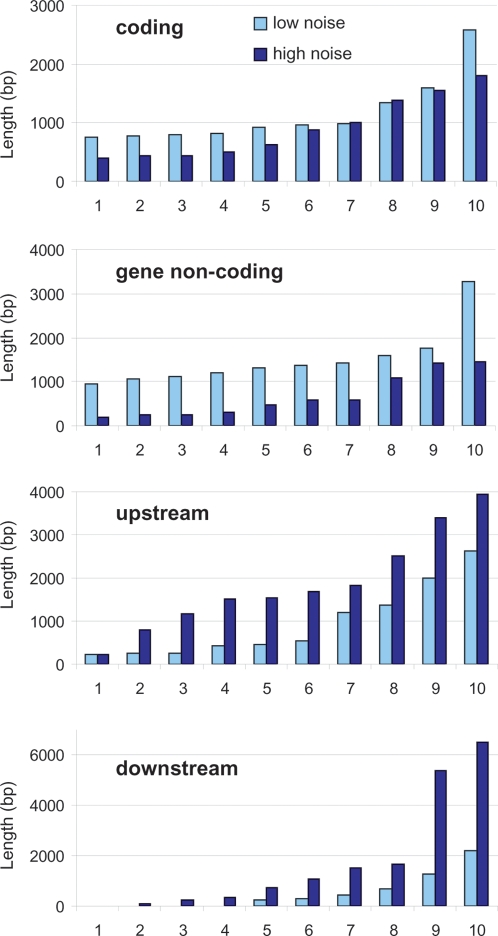
Examples of genetic sequence lengths and expression values for genes with low and high noise. The genome-wide trends that we observe can also be noticed at the level of individual genes, higher noise genes having a tendency for having longer intergenic sequences, shorter gene noncoding and, to a lesser extent, shorter coding sequences. Genes were ordered by increasing noise (replicate variability) value and sets of ten consecutive genes were randomly selected around the ends of the range of values seen in Figure 4E–H. For each category here the values are ordered. The low-noise genes are: AT4G02610, AT5G61580, AT2G36390, AT5G64470, AT5G26210, AT2G45990, AT2G25670, AT2G36530, AT5G66380, AT5G35530, and the high-noise: AT2G45760, AT4G17220, AT1G70830, AT1G29500, AT2G22760, AT4G24700, AT3G13640, AT1G62560, AT1G60870, AT1G68590. See the Supplementary Figure for examples with tissue variability and median expression.

The results presented so far deal with trends over the scale of 10,000 genes. We asked if these trends can also be seen when looking at individual genes. For instance, can we observe that individual genes with higher noise have longer intergenic sequences and shorter genic sequences? To address this question, we randomly selected sets of ten genes with low or high noise, tissue variability, and median expression, and compared the intergenic and gene sequence lengths in the low and high sets. As expected from the relationships in Figure 4, we found that genes with higher noise or tissue variability generally have longer intergenic sequence lengths and smaller gene noncoding sequence lengths, while the contrast is not so clear for the gene coding sequence length, especially for tissue variability (Figure 6 and Figure S1A). For median expression we chose a set at low median (∼1), and another at median ∼3 (about the minimum in Figure 4I, J, and the maximum in Figure 4K, L). Again as expected, genes at median 3 generally have smaller intergenic sequence lengths and longer gene noncoding sequence lengths, with less difference for the coding sequence (Figure S1B). Therefore, the genome-wide trends presented above can also be observed at the scale of individual genes.

## Discussion

Our aim was to perform a thorough and objective study of the relationships between the mRNA expression of genes and the length of their associated noncoding and coding sequences. For this we used tissue-specific microarray data from the *A. thaliana* root, and the *A. thaliana* genome annotation. We considered three aspects of gene expression: tissue variability, expression noise, and median expression between tissues. We first divided sequences between intergenic upstream and downstream and genic coding and noncoding (Figure 1), and obtained the length of each. We found inter-dependencies between the different sequences, and between the gene expression measures (Figure 2 and 3). We then looked at the relationships between length and expression, and found that globally the relationships of intergenic sequences with expression were opposite to that of the genic sequences (Figure 4). Next we looked at the individual components of a gene (the UTRs and the first four introns and coding regions), and found that the noncoding components had opposite relationships to expression compared to the coding components (Figure 5). Finally, we could also observe these genome-wide trends at the level of individual genes (Figure 6).

### Comparison to previous reports

Previous studies have examined some of the relationships between sequence lengths and gene expression, and their results are generally consistent with ours. **Expression variability between tissues**: we found a positive relationship with intergenic sequences. In agreement with this, a positive relationship between intergenic sequence length and expression ‘complexity’ has been reported in *Drosophila melanogaster* and *Caenorhabditis elegans* [4]; human housekeeping genes (generally widely expressed) were found to have shorter intergenic sequences [5]; and finally a positive relationship was found between intergenic sequence length and the breadth of response (number of experiments with differential gene expression) in a recent *A. thaliana* study [6]. The negative relationship between variability and genic sequence length is also similar to that reported in another *A. thaliana* study [6]. Also in accordance with our findings, human genes expressed in all tissues (i.e., with low variability) were found to have shorter introns, UTRs and coding regions [19]. **Median expression**: we found that the relationships with median expression were not linear (Figure 4). Some previous reports suggested that the relationship with expression level was linear and negative [8]–[10], however more recent studies also observed non-linear relationships [6], [12], [20]. To our knowledge, the relationships that we report between **expression noise** and sequence lengths have not been studied previously.

### Contribution of this work

Even though the relationships between sequence lengths and gene expression have been studied in the past by various authors at different depths, here we employed a methodology which differs from previous studies in a number of ways, including: 1) Analyzing all the sequence lengths associated with a gene, i.e. both intergenic and genic; 2) Analyzing, from a single dataset, measures relating to three metrics of gene expression: level (median across tissues), expression diversity across tissues (variability between tissues) and noise (variability between biological replicates). As noted above, the relationships between noise and sequence length had not been studied previously; 3) Making explicit the possible inter-dependencies between the different variables; 4) Using contour plots and smooth regression instead of linear regression or ‘binned data’, i.e. observation methods which do no assume a specific shape for the relationships.

This approach allowed us to make several new observations: 1) Intergenic and genic noncoding sequences globally have opposite relationships with all aspects of gene expression studied, variability between tissues, noise, and level; 2) Expression noise has relationships to sequence lengths that are similar to those of expression variability, and in general these relationships are even stronger than those with variability; 3) The measures for expression noise, expression level and variability that we used here seem to be deeply interconnected with each other, since their inter-interdependencies are in agreement with their relationships with expression.

Even though the genome-wide relationships that we report here are relatively weak, we believe that their biological significance should be taken seriously for the following reasons. First, as noted above, some of our results have been previously reported in other studies, which used other types of datasets and other analysis methods, and animal rather than plant data. Therefore these relationships seem to hold both in plants and animals, strongly arguing for their biological significance. Second, although one should not over-interpret the direction or shape of the local linear regressions since the contour plots show that the data are not uniformly distributed, it is clear that intergenic and genic sequences have opposite relationships with expression, which can be seen even with the contour plots, and it is difficult to imagine how such opposite relationships could arise spuriously.

### Proposed explanation for relationships, based on epigenetic regulation of gene expression

These relationships are not explained by our current understanding of the role of genic noncoding sequences in regulating or influencing gene expression. Taken in isolation, it would appear straightforward to explain the positive relationship between the upstream intergenic sequence length and expression variability. Indeed, since it is known that these sequences harbor regulatory elements important for gene expression, it would be plausible that longer upstream intergenic sequences have a greater potential for harboring a larger number of such regulatory elements, and are therefore able to drive more elaborate expression patterns. This is the explanation proposed by other authors [4], [6]. However, this explanation cannot explain why the length of the gene noncoding sequence also has a relationship with respect to expression variability, and that this relationship is negative (Figure 4). And since intergenic and genic sequences have opposite relationships to expression for all expression measures considered here, it seems more likely that the same causes are involved, at least partially, in creating the relationships with expression of the intergenic and the genic sequences.

It has been suggested earlier that ‘chromosome organization’ could be the source of the relationships between sequence lengths and gene expression [12]. What exactly could be the nature of this organization? It is known that the transcription of a gene can be induced upon re-localization within the nucleus [21], and that cellular differentiation is associated with restriction of chromatin movement on the nuclear matrix [22]. Using our observation that intergenic and genic sequences have opposite relationships with expression, we suggest that intergenic and gene noncoding sequences could have opposite effects on these re-localization and restriction activities, because intergenic sequences are ‘outside’, while gene noncoding sequences are ‘inside’ the transcribed region. It could be that secure attachment of the gene region via the intergenic sequences is required to better control the gene region and to send it to an area of the nucleus where there can be high transcription upon induction. Long gene noncoding sequences could somehow prevent this re-localization, perhaps by keeping the transcribed region securely attached to the matrix, thus lowering the possible variability of expression. Variability of expression of a gene would therefore mostly depend on its ability to be re-localized to a different region of the nucleus upon induction, and this capability would be independent from the overall level at which it can be expressed when it is not under epigenetic regulation. Perhaps the coding sequence does not have much influence on these processes, as suggested by the fact that individual coding sequences have weaker relationships with variability and noise than the noncoding sequence does.

How could this model explain that noise has similar relationships to sequence lengths as expression variability does, and explain why noise and variability are positively related to each other (Figure 3 and 4)? Logically we could think that, on the contrary, a gene which is more variably expressed between tissues should also be regulated epigenetically more ‘tightly’, thus that its expression should be less noisy. However, it is known that the epigenetic state of a DNA region is variable within a population and, unless insulators are present to provide a sharp transition, the change between euchromatin and heterochromatin is gradual [23]. It is therefore possible that, for genes with longer intergenic sequences, because the distance between the regulatory elements which seed the epigenetic markings of the locus is greater, the epigenetic state of the gene is more uncertain.

This model could be tested experimentally in vivo by modifying the sequence lengths of reporters and studying the effects on chromosome structure using chromosome-structure capturing assays [24]. Effects on variability of gene expression across tissues could be accurately measured using newly developed image recognition methods (as in [25]). Overall, our work indicates that studying the relationships between genomic features and gene expression using large-scale gene expression data could help to better understand the relationships between the genome and gene expression.

## Methods

### Genetic annotation data

*Arabidopsis thaliana* genome annotation files available at the TAIR ftp site (ftp://ftp.arabidopsis.org/home/tair/) were parsed with java scripts to obtain genetic sequence components lengths (all but UTRs from: sv_gene_feature.data file, 04/27/06 version; UTRs: TAIR6_3_UTR_20060126 and TAIR6_5_UTR_20060126). The gene noncoding sequence length was calculated by subtracting the coding sequence length from the transcribed region length (because some introns can be in UTRs, this is not necessarily equal to the sum of the lengths of UTRs and introns in a gene, and it gives the accurate value for the ‘total noncoding sequence’ of a gene).

### Transcript expression data

We used microarray data measured in three biological replicates each (except for quiescent center which had two replicates) on the Affymetrix ATH1 GeneChip from the following seven tissues: lateral root cap and epidermis [13]; quiescent center and columella [14]; cortex, xylem and phloem [7]. Microarray data is available at http://www.arexdb.org. Gene expression values were calculated with the MAS5 algorithm (from the Affymetrix software), log(MAS5) values being used. Not shown here, RMA values were also used with slightly different results but not altering our conclusions (see [26]). Let *A_ij_* be the log_10_(MAS5) value in each of 7 tissues, *i*, and 3 replicates (for all but the quiescent center data, 2 replicates), *j*. The mean expression in a tissue and the total mean expression are then:
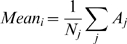


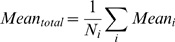
For the expression level we used the median across tissues:
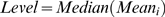
Between-tissue and within-tissue variances were obtained by fitting the random-effects model A_−ij_ = α+β_i_+ε_ij_ for each gene via restricted maximum likelihood [27], where β_i_'s are tissue random-effects with variance 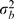, and where ε_ij_'s have variance σ^2^. The estimated variance components
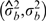
provide the gene-specific between-tissue variability and noise variability, respectively. Estimation was performed using the *lme* package in R [28].

### Data set construction and analysis

Of 28,580 annotated genes in the *A. thaliana* genome, we retrieved 11,725 with both expression information and annotation (data table available in Table S1). Genes annotated such that the gene length was inferior to the coding sequence length or which did not have an annotation for the coding sequence length were discarded. Only the first listed alternative transcript of each gene was considered. When the adjacent gene overlapped, the intergenic sequence length was put to zero. When a UTR was not annotated, its length was put to 0. For each graph, zero values of sequence length were discarded. Data analysis was performed in R. Contours were obtained from a 2 d kernel density estimate; zero lengths are not shown. The solid red line shows the trend in y axis as a function of x axis obtained via a local linear regression smoother. Dashed red lines show 95% confidence intervals, blue lines show constant mean and axes are scaled to the (0.5%, 0.95%) quantiles of the respective variables.

## Supporting Information

Figure S1 Examples of genetic sequence lengths and expression values for genes with low and high variability and median ∼1, median ∼3. Genes were ordered by increasing variability (A) or median (B) value, and sets of ten consecutive genes were randomly selected around the ends of the ranges of values seen in Figure 4A–D for variability (A), and at low median (∼1) or about peak median (∼3) (B). For each category here the values are ordered. The low variability genes are: AT1G79900, AT3G03320, AT4G26240, AT2G36590, AT3G05760, AT3G58800, AT1G08980, AT1G08710, AT5G42190, AT2G20860, and the high variability: AT2G31085, AT5G19530, AT4G22212, AT2G38170, AT2G23760, AT3G29770, AT1G23410, AT4G38080, AT1G61380, AT1G80240. The low median: AT3G27810, AT1G63150, AT5G25610, AT2G43580, AT2G33810, AT5G65870, AT4G03060, AT3G10570, AT5G45670, AT1G62060, and the high median: AT3G01070, AT1G22190, AT1G14910, AT1G10130, AT1G78150, AT2G25970, AT1G63220, AT1G25380. AT1G53400, AT1G79870.
(1.21 MB EPS)

Table S1 Data file used for the analysis.
(1.72 MB TXT)
